# Early side effects and first results of radioligand therapy with ^177^Lu-DKFZ-617 PSMA of castrate-resistant metastatic prostate cancer: a two-centre study

**DOI:** 10.1186/s13550-015-0114-2

**Published:** 2015-06-20

**Authors:** Hojjat Ahmadzadehfar, Kambiz Rahbar, Stefan Kürpig, Martin Bögemann, Michael Claesener, Elisabeth Eppard, Florian Gärtner, Sebastian Rogenhofer, Michael Schäfers, Markus Essler

**Affiliations:** Department of Nuclear Medicine, University Hospital Bonn, Sigmund-Freud-Str. 25, 53127 Bonn, Germany; Department of Nuclear Medicine, University Hospital Muenster, Muenster, Germany; Department of Urology, University Hospital Muenster, Muenster, Germany; Department of Urology, University Hospital Bonn, Bonn, Germany; European Institute for Molecular Imaging, University of Muenster, Muenster, Germany

**Keywords:** Prostate cancer, PSMA, Radioligand therapy, 177Lu

## Abstract

**Background:**

Radioligand therapy (RLT) with ^177^Lu-DKFZ-617 PSMA (Lu-PSMA) (prostate-specific membrane antigen) is a novel targeted therapy of metastatic prostate cancer. We analysed retrospectively the early side effects and the response rate in the first patients, who received a therapy with Lu-PSMA in our departments.

**Methods:**

RLT was performed in ten hormone- and/or chemo-refractory patients with distant metastases and progressive disease (mean age 73.5 years). ^68^Ga-PSMA HBED-CC PET/CT was performed in all patients prior to RLT. The median PSA level prior to the therapy was 298.5 ng/ml (range 5–853 ng/ml). All patients received CBC, renal and liver function tests the day before and 2 days after application (mean administered activity 5.6 GBq, range 4.1–6.1 GBq), followed by further tests every 2 weeks. All patients were contacted by telephone every week regarding side effects or any positive and negative changes.

**Results:**

Eight weeks after the therapy, seven patients (70 %) experienced a PSA decline, of whom six experienced more than 30 % and five more than 50 %. Three patients showed a progressive disease according to the PSA increase. No patient experienced any side effects immediately after injection of Lu-PSMA. Relevant hematotoxicity (grade 3 or 4) occurred 7 weeks after the administration in just one patient. The same patient showed a leucopenia grade 2. Two patients showed a disturbance of only 1 hematologic cell line, whereas one patient showed a reduction of grades 1 and 2 in leucocytes and thrombocytes, respectively. Six patients did not show any hematotoxicity during the 8 weeks after therapy. There was no relevant nephrotoxicity (grade 3 or 4).

**Conclusions:**

Our initial results indicate that RLT with Lu-PSMA is safe and seems to have low early side-effect profile. A relevant PSA decline was detected in 70 % of patients.

## Background

Prostate cancer (PCa) is the second most common cancer worldwide in male and the fourth most common cancer overall [1]. In patients with localised PCa, the five-year survival rate approximates 100 %; however, in patients with distant metastases, the five-year survival rate drops to 31 % [2]. Almost all patients with metastatic PCa will initially respond to well-established and innovative anti-androgen treatments including the two recently approved hormone therapy agents, the androgen receptor antagonist enzalutamide and the CYP17A1-inhibitor abiraterone [3, 4] which significantly improve the overall survival. However, progression to androgen independence is the main cause of death in prostate cancer patients [5]. Most deaths related to PCa are due to advanced disease, which results from any combination of lymphatic, blood, or contiguous local spread. Targeted radionuclide therapy is an attractive and quickly developing therapy option for many different cancers, such as lymphoma, melanoma, and neuroendocrine tumours [6–9]. Prostate-specific membrane antigen (PSMA) is highly expressed on prostate epithelial cells and strongly up-regulated in prostate cancer. The PSMA expression levels are directly correlated to androgen independence, metastasis, and PCa progression [10]; therefore, PSMA is an attractive target for diagnosis and therapy of metastasised prostate cancer. After rather unsuccessful therapy with ^90^Y-CYT-356 monoclonal antibody (mAb) that binds to the intracellular domain of PSMA [11], phases 1 and 2 clinical trials utilising the PSMA mAb J591, radiolabelled with ^90^Y or ^177^Lu, have shown promising results [12–15]. Recently, a novel theranostic drug, ^177^Lu-DKFZ-617, which is a DOTA derivative of the Glu-urea-Lys motif, has been developed in the Department of Nuclear Medicine, University Hospital Heidelberg, Germany, for the treatment of patients with metastatic prostate cancer [16, 17]. In this study, we analysed retrospectively the early side effects and the response rate in the first patients, who received a therapy with ^177^Lu-DKFZ-617 PSMA (Lu-PSMA), in our departments as the last possible option [18].

## Methods

Ten consecutive hormone- and/or chemo-refractory PCa patients with distant metastases and progressive disease according to the PSA level were treated with Lu-PSMA between November 2013 and January 2014 in two different centres (University Hospital Bonn and University Hospital Muenster). All patients underwent a ^68^Ga-PSMA HBED-CC (^68^Ga-PSMA) PET/CT prior to therapy to evaluate the PSMA expression status of the metastases. The majority of patients had bone metastases as well as lymph node metastases. The extent of metastases in the patients is shown in Table 1. The written information regarding the therapy and its possible side effects was provided to each patient at two time points: first after the PET scan and once again 24 h prior to therapy. The local committee on ethics approved this retrospective study, and all subjects had provided prior written informed consent.

**Table 1:** The extent of metastases and history of chemotherapy in ten patients with prostate cancer

Number	Age (y/o)	Metastases	History of CTx	Dose GBq
1	70	Bone3, Ln1, LR	No	5.8
2	77	Bone3, LR	Yes	6.1
3	67	Bone3, Ln1, LR	Yes	6.0
4	75	Bone3, Ln1	No	5.9
5	77	Bone3, Ln2	No	4.1
6	81	Bone3, Ln1	No	5.9
7	62	Ln2	Yes	5.8
8	74	Ln2, liver met	Yes	5.7
9	76	Bone1, Ln1,	Yes	5.8
10	76	Bone3, Ln1	No	5.2

Bone1 <6 bone metastases, bone2 6–20 bone metastases, bone3 >20 bone metastases

*Ln1* lymph node metastasis (abdominal, iliacal and inguinal), *Ln2* Ln1 + thoracal lymph node metastasis, *LR* local recurrence, *CTx* chemotherapy

### Treatment planning

#### ^68^Ga-PSMA HBED-CC PET/CT

^68^Ga-PSMA was applied by a slow intravenous injection (30–60 s) using a weight-adapted dose of 2 MBq/kg body weight in a total volume of 5–10 ml (diluted with 0.9 % sterile sodium chloride solution), followed by 20 ml of sterile 0.9 % sodium chloride. The average injected dose was 140 MBq. PET/CTs were performed on a Biograph 2 PET/CT scanner in Bonn (Siemens Medical Solutions, Erlangen, Germany) and on a Biograph mCT (Siemens Medical Solutions, Erlangen, Germany) in Muenster. Depending on the clinical situation and the availability of previous CT examinations, either a diagnostic CT including the application of intravenous contrast media or a low-dose CT without contrast agent was performed.

#### Renal function test and renal scintigraphy with ^99m^Tc-MAG3

Creatinine and glomerular function tests (GFR) were performed in all patients prior to the therapy and every 2 weeks afterwards. To rule out any renal obstructive disease as well as for the measurement of the tubular extraction rate of MAG3 (TER MAG3), all patients underwent a renal perfusion scintigraphy with ^99m^Tc-MAG3 within 1 week prior to and 8 weeks after the therapy. The scans were performed using dual-head SPECT cameras (AnyScan, Mediso at the University Hospital Bonn and Siemens E-Cam at the University Hospital Muenster). This procedure has been described in detail elsewhere [19, 20].

#### Salivary gland scintigraphy with ^99m^Tc-Pertechnetate

To evaluate the functional impairment of the salivary glands, all patients received a dynamic salivary gland scintigraphy with Tc-Pertechnetate combined with salivary gland stimulation by lemon juice 20 min p.i. on the treatment day as well as 8 weeks after the therapy. The scans were performed on a triple-head SPECT camera (Irix Philips) at the University Hospital Bonn and on a dual-head SPECT camera (Siemens E-Cam) at the University Hospital Muenster. The scintigraphic procedure has been described in detail elsewhere [21].

### Radioligand therapy (RLT)

PSMA was obtained from ABX GmbH (Radeberg, Germany). To begin, 1 mg DOTA-PSMA was dissolved in 1 ml 0.05 M HCl. Then, 88.50 ± 9.21 μg DOTA-PSMA per 10 μg Lu was added to 1 ml 0.05 M HCl solution containing 42 mg gentisinic acid and 210 mg sodium ascorbate. This mixture was added to carrier-added ^177^LuCl_3_, obtained from IDB Holland, and heated for 30 min at 95 °C. Quality control was performed by spotting 1 μl aliquots on TLC (SilicaGel 60, Merck, Darmstadt, Germany) with 0.1 M citric buffer or ITLC-SG plates (ITLC-SG, Varian, Lake Forest, CA, USA) and with 1 M NH_4_OAc/MeOH (1:1) as solvent. Analysis was performed using a flat-bed scanner (Rita Star, Raytest-Isotopenmessgeräte GmbH, Straubenhardt, Germany). Radiochemical purity was determined by radio HPLC, which was performed using a gradient system. The gradient elution system utilised mobile phase A (deionised H_2_O containing 0.1 % TFA) and mobile phase B (100 % acetonitrile) and a flow rate of 1.0 ml/min. Starting with 100 % A/0 % B, the gradient was increased to 100 % B over 30 min and then returned to the initial gradient conditions within 5 min. The retention time of free ^177^Lu was *R*_t_ = 2.5 min, whereas for Lu-PSMA it was 13.3 min.

The labelling yield always exceeded 95 % (98.85 ± 1.29 %); therefore, no purification was performed. The radiochemical purity was higher than 98 %. The specific activity of Lu-PSMA was 89.73 ± 13.61 MBq/μg.

The therapy solution was administered by slow intravenous injection over 1 min followed by 1000 ml of NaCl or Ringer. In order to reduce therapy-induced damage to the salivary glands, the patients received ice packs over the parotid and submandibular glands from 30 min prior to and up to 4 h after administration of the Lu-PSMA. All patients were discharged 48 h after therapy according to the rules of the Federal Office for Radiation Protection in Germany (BfS).

### Data collection and follow-up

One day prior to and 2 days after therapy, the haematological and renal status, liver function tests, tumour marker PSA, alkaline phosphatase, and blood biochemistry were evaluated in all patients. The ECOG performance-status score, the therapy-induced side effects during the time of hospitalisation and at follow-up, and laboratory examinations at two-week intervals for up to 8 weeks after therapy were obtained in all patients. All patients were contacted by telephone regularly in one- to two-week intervals. The blood examinations were conducted by the urologists and faxed to our departments.

### Tumour response evaluation

The tumour marker PSA was used as the main marker for the response evaluation. We classified the changes of PSA level as decreasing more than 50 %, more than 30 %, and any decline. An increase of ≥25 % was evaluated as progress.

### Toxicity

Toxicity was recorded with the Common Terminology Criteria for Adverse Events (CTCAE), version 4.0, and was analysed according to the recommendation of NCI guidelines for investigators.

## Results

Ten hormone- and/or chemo-refractory patients with a mean age of 73.5 years (range 62–81) underwent ^68^Ga-PSMA PET/CT followed by therapy with a mean of 5.6 GBq Lu-PSMA (range 4.1–6.1 GBq). All patients had a history or were under therapy with enzalutamide and/or abiraterone. Four patients had received ^223^Ra-dichto chloride (1–4 cycles). Eight patients showed PSMA-positive bone and lymph node metastases, from whom seven had a massive metastatic disease with more than 20 bone metastases (Table 1). Three patients (patients 1–3; Table 1) also showed local recurrence. One patient had only lymph node metastases (patient 7; Table 1) and one had a liver metastasis along with lymph node metastases (patient 8; Table 1). The mean and median PSA levels prior to the therapy were 339.4 and 298.5 ng/ml, respectively (range 5–853 ng/ml).

Nine patients exhibited good Eastern Cooperative Oncology Group (ECOG) performance-status scores (0 or 1; Table 2). The blood parameters are shown in Table 3.

**Table 2:** Performance status and baseline PSA level as well as PSA changes 8 weeks after therapy

Number	ECOG1	ECOG2	Baseline PSA (ng/ml)	PSA2 (ng/ml)	PSA change (%)
1	1	1	226	529	134.7
2	1	1	334	243	−27.2
3	2	2	376	141	−62.5
4	1	1	853	590^a^	−30.8
5	1	1	148	46	−68.9
6	0	0	20	5	−75.0
7	1	1	263	355	35.0
8	1	1	790	293	−62.9
9	1	1	379	58	−84.7
10	3	2	5	21	320.0

*ECOG1* baseline Eastern Cooperative Oncology Group performance status, *ECOG2* Eastern Cooperative Oncology Group performance status 2 months after therapy, *PSA* prostatic specific antigen, *PSA2* prostatic specific antigen 2 months after therapy

^a^4 weeks after Tx, PSA decreased to 71 ng/ml

**Table 3:** Hb, WBC and Plt values on the treatment day and hematotoxicity measured 2 months after Lu-PSMA therapy

Number	Baseline Hb (g/dl)	CTC Hb	Baseline WBC (g/l)	CTC WBC	Baseline Plt (g/l)	CTC Plt
1	9.6	0	7.4	0	352	0
2	11	0	7.1	0	322	0
3	9.0	0	4.7	0	181	1
4	11.7	3	3.3	2	62	0
5	11.5	0	5.9	0	170	0
6	14.5	0	8.8	0	234	0
7	11.4	0	8.0	0	295	0
8	12.2	1	10.1	0	313	0
9	9.3	0	3.4	1	99	2
10	9.9	0	7.3	0	321	0

Patient 4, 9 and 10 had received blood transfusion prior to therapy because of low Hb levels

*CTC* common toxicity criteria 2 months after therapy, *Hb* hemoglobulin, *PLt* platelet, *WBC* white blood cells

Two patients (patient 4 and 10) had received blood transfusion prior to therapy because of grade 3 anaemia, and patient 9 had received blood transfusion because of bleeding in the bladder, which consisted till 7 weeks after the therapy.

### Response evaluation 2 months after therapy

**Fig. 1:**
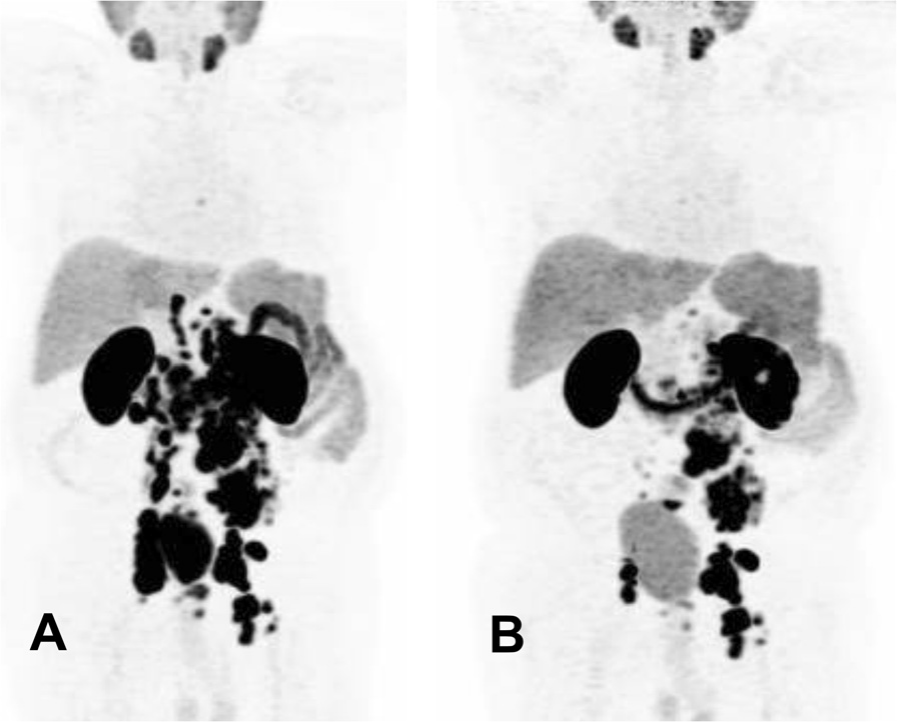
A 74-year-old patient with hormone- and chemo-refractory prostate cancer underwent PSMA PET/CT (**a**), which showed diffuse abdominal and iliacal lymph node metastases. The patient underwent RLT with 5.7 GBq Lu-PSMA. The PSA level was at the time of the therapy 790 ng/ml. **b** A partial response 7 weeks after RLT with 63 % PSA decline; at this time, the PSA level was 293 ng/ml

Eight weeks after the therapy, seven patients (70 %) experienced a PSA decline, out of whom six experienced more than 30 % and five more than 50 %. Three patients showed a progressive disease according to the PSA increase (Table 2, Fig. 1).

### Complaints and side effects during hospitalisation

No patient experienced any side effects immediately after injection of Lu-PSMA. No significant change in blood pressure or body temperature was observed. One patient complained of a light headache 1 day after application (patient 2). The pain was exactly in the region of his bone metastasis on the skull. It was treated successfully with one 400-mg dose of ibuprofen. The same patient had dry lips beginning 2 days after application and lasting for 2 weeks. One patient experienced mild nausea (patient 10), and one (patient 4) with a history of intermittent nausea experienced nausea and one instance of vomiting about 6 h after administration of the Lu-PSMA. In both patients, the nausea was controlled easily with ondansetron. Otherwise, the patients tolerated the therapy very well.

### Complaints and side effects between the second day and 2 months after therapy

Two patients complained of fatigue and two other patients complained of hypogeusia and dry lips in the first 2 weeks (Table 4). Patient 4 experienced fatigue 7 weeks after the therapy due to anaemia (Hb 6.9), which was treated with 2 units of packed red blood cells (pRBC). No patient experienced a negative change in performance status (Tables 2 and 4).

**Table 4:** Side effects and complaints in the first 2 days, 4 weeks and between 4 and 8 weeks

Number	In the first 2 days	During the first 4 weeks	4–8 weeks
1	No	No	No
2	Dry lips	Dry lips just in the first 2 weeks	No
	Light headache		
3	No	No	No
4	Nausea and vomiting just 1 time	No	Fatigue in the 7th week because of anaemia, patient received red blood cell transfusion
5	No	Hypogeusia in the first 2 weeks	No
6	No	No	No
7	No	No	No
8	No	No	Fatigue
9	No	No	No
10	Nausea	No	No

The follow-up salivary gland scintigraphy 2 months after therapy showed no significant change in the uptake and clearance of ^99m^Tc from the salivary glands.

### Hematotoxicity

The mean Hb, WBC, and Plt prior to therapy were 11.0, 6.6, and 234.9 g/l, respectively (Table 3).

Relevant hematotoxicity (grade 3 or 4) occurred 7 weeks after the administration in just one patient, who had a history of blood transfusion prior to therapy (patient 4). The same patient showed a leucopenia grade 2. Two patients showed a disturbance of only 1 hematologic cell line (thrombocytopenia, 1; anaemia, 1), whereas one patient showed a reduction of grades 1 and 2 in leucocytes and thrombocytes, respectively. Six patients (60 %) did not show any hematotoxicity during the 8 weeks after therapy (Table 3).

### Nephrotoxicity

There was no relevant nephrotoxicity (grade 3 or 4). One patient showed a grade 1 and one with a known renal insufficiency showed a grade 2 toxicity (Table 5).

**Table 5:** Nephrotoxicity

Number	Cr	CTC Cr	GFR (ml/min)	CTC GFR	Baseline TER MAG3	Second TER MAG3
1	1.0	1	>70	1	183	179
2	1.3	0	56	0	125	104
3	0.8	0	>70	0	245	209
4	0.6	0	>70	0	186	178
5	1.5	2	50	2	92	96
6	0.7	0	>70	0	208	220
7	0,6	0	>70	0	270	284
8	1,2	0	>70	0	156	220
9	0,8	0	>70	0	214	214
10	0,9	0	>70	0	231	256

*Cr* creatinine, *GFR* glomerular filtration rate, *TER MAG3* tubular extraction rate of MAG3 (ml/min/1.73 m^2^ BSA)

### Hepatic toxicity

There was no toxicity except for one grade 1 hypoalbuminemia (Table 6).

**Table 6:** Liver toxicity

Number	CTC ALT	CTC AST	CTC albumin
1	0	0	0
2	0	0	0
3	0	0	0
4	0	0	0
5	0	0	1
6	0	0	0
7	0	0	0
8	0	0	0
9	0	0	0
10	0	0	0

*ALT* alanine aminotransferase, *AST* aspartate aminotransferase, *CTC* common toxicity criteria

## Discussion

Targeted radionuclide therapy is a state of the art and rapidly developing therapy option for different cancer types. The potential advantage of targeted radionuclide therapy is saving the normal tissue while giving a high radiation dose to the tumour. PSMA is highly overexpressed by almost all prostate cancer cells; hence, it is an optimal target for radionuclide therapy. Tagawa *et al.* presented the results of a phase II study of radionuclide therapy with Lu-PSMA mAb J591 [12], which was based on two former phase I-published studies with this agent [13, 22]. mAb are large molecules and therefore show a poor permeability in solid tumours and slow clearance from the circulation. This combination leads to suboptimal tumour targeting and an increased absorbed dose to red marrow, narrowing the therapeutic window [23]. Maresca *et al.* described the design and synthesis of a series of small molecule inhibitors of PSMA [24]. On the basis of this work, Hiller *et al.* [17] did the preclinical evaluation of two radiopharmaceuticals, ^123^I-MIP-1072 and ^123^I-MIP-1095, that were designed to target PSMA in prostate cancer cells and tissue. In a very recent published study from the Heidelberg group, Zechmann *et al.* showed the utility of ^131^I-MIP-1095 PSMA [23]. In contrast to mAb, the low molecular weight compounds, with higher permeability into solid tumours, offer a significant advantage in achieving higher uptake per gramme of tumour tissue and a high percentage of specific binding. Furthermore, small molecules display more rapid tissue distribution and faster blood clearance compared with intact immunoglobulins. These properties often lead to an enhanced target to non-target tissue ratio, which is important not just for imaging but also for successful application of therapeutic absorbed doses [23]. ^131^I has a half-life of 8.02 days with a high environmental radiation burden according to the high gamma energy, which limits its utility because of complex radiation protection regulations in most countries. In contrast to ^131^I, ^177^Lu causes a lower local dosis rate, and therefore a lower radiation burden for staff and contact persons. Furthermore, the higher specific activity of ^177^Lu compared to commercially available ^131^I makes ^177^Lu preferable for targeted radionuclide therapies. Late renal toxicity of ^177^Lu-PSMA is subject of further studies.

In this paper, we introduced the first experiences with Lu-PSMA in our departments for the treatment of metastatic prostate cancer. Our patients did not have any other therapy option and were selected very carefully for this therapy in cooperation with their urologists or oncologists. The first and the most important issue for us was the tolerability of the therapy regarding incidence of post therapy symptoms as well as toxicities. Our second aim was to evaluate the therapy response after a single dose application of this agent.

The tolerability was very good, and no patient experienced any serious side effects in the time of hospitalisation or afterwards.

Tagawa *et al.* [12] treated 47 patients with Lu-PSMA mAb J591. In their study, a total of 10.6 % experienced >50 % decline in PSA, 36.2 % experienced >30 % decline, and 59.6 % experienced any PSA decline following their single treatment. All patients experienced reversible hematologic toxicity, with grade 4 thrombocytopenia occurring in 46.8 % (29.8 % received platelet transfusions) without significant haemorrhage. A total of 25.5 % experienced grade 4 neutropenia. Zechmann *et al.* [23] reported a decline in serum PSA levels of ≥50 % in 60.7 % of patients and a drop between 0 and 50 % after a single dose of ^131^I-MIP-1095 PSMA in 14.2 % of patients. In our study 70 % of the patients showed a decline in PSA level, with 50 % of them at more than 50 %. Here, we have to mention that we introduce the preliminary results of our therapies in this paper, and comparison of the results of our study with these two studies is not justified for many reasons, including the different types of the studies (prospective vs. retrospective), the number of patients, and the duration of follow-up.

In most cases there was extensive skeletal and lymph node involvement, and a majority of patients were clinically anaemic at the time of treatment; however, a mild hematotoxicity was observed in just three patients. In the time of Lu-PSMA therapy, the Hb value of patient 4, who had a history of four cycles of ^223^Ra therapy, was 11.7 g/dl, and therefore, we classified his Hb decline 7 weeks after therapy as grade 3 toxicity. This patient also suffered from a chronic thrombocytopenia after four cycles of therapy with ^223^Ra, and the decision for the Lu-PSMA therapy was made after a detailed clarification of all possible side effects. The number of thrombocytes did not change in the follow-up time.

Because of the physiological PSMA expression in the kidneys, there is concern regarding potential toxicity due to radiation to the kidneys. Zechmann *et al.* [23] reported after therapy with ^131^I-MIP-1095 PSMA that there was no apparent evidence or negative trend in either calculated GFR or serum creatinine levels in a one-year follow-up. In our study, no patient experienced a grade 3 nephrotoxicity, and one patient with known renal insufficiency showed a grade 2 nephrotoxicity and one patient a grade 1. Apart from blood GFR and creatinine, renal scintigraphy with Tc-MAG3 is a valuable examination prior to therapy to rule out any relevant obstructive disease. An obstructive disease should be treated in advance for reducing the delivered dose to the affected kidney. We have to mention here that after a two-month follow-up, we can rule out acute renal insufficiencies, but we have to do a longer follow-up to determine any chronic side effects.

Regarding xerostomia, because of the physiologic uptake of this tracer in salivary glands, the side effects were mild and transient and occurred in just two patients. We do not know for certain whether a cooling of the salivary glands is an effective therapy for saving these glands from radiation; however, using it is tolerable for the patients and is not harmful. There is a need to plan a PET study regarding this issue measuring the change in uptake of ^68^Ga-PSMA with and without ice packs.

As mentioned before, this paper presents our first experiences with Lu-PSMA, and we are going to increase our knowledge about this therapy. There are some important issues which should be studied in the future, such as the number of cycles, the maximal tolerable dose, and the quality of life of these patients. One of the most important questions is whether there is a positive effect on overall survival, which needs a long follow-up time.

## Conclusions

Our initial results indicate that a single dose of Lu-PSMA for the treatment of metastatic prostate cancer patients without any other therapy option is safe and seems to have a low early side-effect profile with evidence of positive response to the therapy according to PSA decline in 70 % of patients.

## References

[1] Ferlay J, Soerjomataram I, Dikshit R, Eser S, Mathers C, Rebelo M (2015). Cancer incidence and mortality worldwide: sources, methods and major patterns in GLOBOCAN 2012. Int J Cancer.

[2] Jemal A, Siegel R, Xu J, Ward E (2010). Cancer statistics, 2010. CA Cancer J Clin.

[3] Scher HI, Fizazi K, Saad F, Taplin ME, Sternberg CN, Miller K (2012). Increased survival with enzalutamide in prostate cancer after chemotherapy. N Engl J Med.

[4] Ryan CJ, Smith MR, Fizazi K, Saad F, Mulders PF, Sternberg CN (2015). Abiraterone acetate plus prednisone versus placebo plus prednisone in chemotherapy-naive men with metastatic castration-resistant prostate cancer (COU-AA-302): final overall survival analysis of a randomised, double-blind, placebo-controlled phase 3 study. Lancet Oncol.

[5] Wei Q, Li M, Fu X, Tang R, Na Y, Jiang M (2007). Global analysis of differentially expressed genes in androgen-independent prostate cancer. Prostate Cancer Prostatic Dis.

[6] Kraeber-Bodere F, Bodet-Milin C, Rousseau C, Eugene T, Pallardy A, Frampas E (2014). Radioimmunoconjugates for the treatment of cancer. Semin Oncol.

[7] Mier W, Kratochwil C, Hassel JC, Giesel FL, Beijer B, Babich JW (2014). Radiopharmaceutical therapy of patients with metastasized melanoma with the melanin-binding benzamide 131I-BA52. J Nucl Med.

[8] van der Zwan WA, Bodei L, Mueller-Brand J, de Herder WW, Kvols LK, Kwekkeboom DJ (2015). GEPNETs update: radionuclide therapy in neuroendocrine tumors. Eur J Endocrinol.

[9] Bodei L, Kidd M, Paganelli G, Grana CM, Drozdov I, Cremonesi M (2015). Long-term tolerability of PRRT in 807 patients with neuroendocrine tumours: the value and limitations of clinical factors. Eur J Nucl Med Mol Imaging.

[10] Santoni M, Scarpelli M, Mazzucchelli R, Lopez-Beltran A, Cheng L, Cascinu S (2014). Targeting prostate-specific membrane antigen for personalized therapies in prostate cancer: morphologic and molecular backgrounds and future promises. J Biol Regul Homeost Agents.

[11] Deb N, Goris M, Trisler K, Fowler S, Saal J, Ning S (1996). Treatment of hormone-refractory prostate cancer with 90Y-CYT-356 monoclonal antibody. Clin Cancer Res.

[12] Tagawa ST, Milowsky MI, Morris M, Vallabhajosula S, Christos P, Akhtar NH (2013). Phase II study of Lutetium-177-labeled anti-prostate-specific membrane antigen monoclonal antibody J591 for metastatic castration-resistant prostate cancer. Clin Cancer Res.

[13] Bander NH, Milowsky MI, Nanus DM, Kostakoglu L, Vallabhajosula S, Goldsmith SJ (2005). Phase I trial of 177lutetium-labeled J591, a monoclonal antibody to prostate-specific membrane antigen, in patients with androgen-independent prostate cancer. J Clin Oncol.

[14] Vallabhajosula S, Goldsmith SJ, Hamacher KA, Kostakoglu L, Konishi S, Milowski MI (2005). Prediction of myelotoxicity based on bone marrow radiation-absorbed dose: radioimmunotherapy studies using 90Y- and 177Lu-labeled J591 antibodies specific for prostate-specific membrane antigen. J Nucl Med.

[15] Vallabhajosula S, Goldsmith SJ, Kostakoglu L, Milowsky MI, Nanus DM, Bander NH (2005). Radioimmunotherapy of prostate cancer using 90Y- and 177Lu-labeled J591 monoclonal antibodies: effect of multiple treatments on myelotoxicity. Clin Cancer Res.

[16] Kratochwil C, Giesel FL, Eder M, Afshar-Oromieh A, Benesova M, Mier W *et al.* [Lu]Lutetium-labelled PSMA ligand-induced remission in a patient with metastatic prostate cancer. Eur J Nucl Med Mol Imaging. 2015. doi:10.1007/s00259-014-2978-1. 10.1007/s00259-014-2978-1 25573634

[17] Hillier SM, Maresca KP, Femia FJ, Marquis JC, Foss CA, Nguyen N (2009). Preclinical evaluation of novel glutamate-urea-lysine analogues that target prostate-specific membrane antigen as molecular imaging pharmaceuticals for prostate cancer. Cancer Res.

[18] National Cancer Institute Guidelines For Investigators (2013). Adverse event reporting requirements for DCTC DCTD (CTEP and CIP) and DCP INDs and IDEs.

[19] Zajic T, Moser E (2004). Procedure guidelines for dynamic renal scintigraphy. Nuklearmedizin.

[20] Bubeck B, Brandau W, Weber E, Pomer S, Georgi P (1990). zum Winkel K. Renal function studies using 99mTc-MAG3: pharmacokinetics and slope clearance determination. Contrib Nephrol.

[21] MacDonald A, Burrell S (2009). Infrequently performed studies in nuclear medicine: part 2. J Nucl Med Technol.

[22] Milowsky MI, Nanus DM, Kostakoglu L, Vallabhajosula S, Goldsmith SJ, Bander NH (2004). Phase I trial of yttrium-90-labeled anti-prostate-specific membrane antigen monoclonal antibody J591 for androgen-independent prostate cancer. J Clin Oncol.

[23] Zechmann CM, Afshar-Oromieh A, Armor T, Stubbs JB, Mier W, Hadaschik B (2014). Radiation dosimetry and first therapy results with a (124)I/ (131)I-labeled small molecule (MIP-1095) targeting PSMA for prostate cancer therapy. Eur J Nucl Med Mol Imaging.

[24] Maresca KP, Hillier SM, Femia FJ, Keith D, Barone C, Joyal JL (2009). A series of halogenated heterodimeric inhibitors of prostate specific membrane antigen (PSMA) as radiolabeled probes for targeting prostate cancer. J Med Chem.

